# Influence of Resilience, Everyday Stress, Self-Efficacy, Self-Esteem, Emotional Intelligence, and Empathy on Attitudes toward Sexual and Gender Diversity Rights

**DOI:** 10.3390/ijerph17176219

**Published:** 2020-08-27

**Authors:** Francisco Manuel Morales Rodríguez, René Rodríguez Clares, María Remedios García Muñoz

**Affiliations:** 1 Department of Educational and Developmental Psychology, Faculty of Psychology, University of Granada, Campus Universitario de Cartuja, 18071 Granada, Spain; 2 Inserta Andalusia, Faculty of Psychology, University of Granada, Campus Universitario de Cartuja, 18071 Granada, Spain; carmenclares@correo.ugr.es; 3 Interdisciplinary Studies of Women Seminar, Faculty of Philosophy and Letters, University of Malaga, Teatinos Campus, 29071 Malaga, Spain; remediosgarmu@gmail.com

**Keywords:** attitudes toward sexual orientation and gender identity rights, university students, resilience, everyday stress, emotional intelligence, burnout, empathy

## Abstract

The present study forms part of the project “Cross-disciplinary education for sexual, body, and gender diversity” (Code 419). The aim of this study was to analyze the role played by the psychoeducational variables involved in burnout (resilience, self-efficacy, self-esteem, emotional intelligence, empathy, and everyday stress) on attitudes toward sexual and gender diversity rights. Participants comprised 170 university students undertaking a degree in primary education. Instruments were administered to assess the constructs analyzed, ensuring informed consent, voluntary participation, anonymity, and data confidentiality. An ex post facto design was employed to determine whether attitudes toward sexual and gender diversity rights are influenced by the possible relationships and role of these variables. We found statistically significant associations between students’ attitudes toward sexual and gender diversity at all three levels (sociocultural, relational, and personal) and the variable of burnout. Attitudes towards gender sexual orientation and gender identity rights influence burnout, and vice versa. As we ponder deeply about how these factors influence one another, we can shift our perspectives in a way that builds social harmony. It is important to learn how exactly these influences work, and this knowledge translates into making teaching strategies more effective to help raise awareness about guaranteeing rights for all. At the personal level of students’ attitudes toward sexual and gender diversity/equality, we found positive correlations between this level and the total score for the variable of resilience and with its factor of personal competence. The data obtained will be of use for future psychoeducational assessment and intervention programs related to an education in sexual orientation and gender identity rights that are aimed at developing socio-emotional competencies and attention to diversity with the ultimate goal of improving social harmony by dismantling stereotypes and raising awareness of the importance of the variables of resilience, self-efficacy, self-esteem, emotional intelligence, empathy, and everyday stress which highlights how “education is an instrument of social transformation”.

## 1. Introduction

The construction of citizen values entails guaranteeing rights in equal conditions for gay, lesbian, trans, transsexual, intersex and queer persons. The fight for equal opportunities between genders associated with optimal mental health in the university and professional work worlds regardless of sex-affective orientation remains a challenge for transforming legal into real equality. The sexist ideological model and hostile behavior towards LGBTIQ (lesbian, gay, bisexual, transsexual, intersex and queer) people that differs from the heteronormative model must be seen as a consequence of the patriarchal system and heterocentrism.

There is a growing interest in assessing attitudes towards sexual and gender equality and diversity and the relationships of the variables that can influence them. However, there are still few university and workplace studies that focus on examining such attitudes and their relationship to variables such as emotional intelligence, empathy, stress, and resilience as is done in this study.

There is an increasing number of legislative provisions to make these human rights effective (Law 23/2018, November 29, on the equality of LGBT people. BOE (Official Gazette) Nr. 10 Friday 11 January 2019 of the Valencian Community [1]; Law 8/2017, December 28, to guarantee the rights, equal treatment and nondiscrimination of LGBT people and their families in Andalusia. (Art. 17) [2]; Protection by sexual orientation: Council of Europe and the European Union (e.g., Rivas-Vañó, [3]), etc.

It is also the goal number five of the 2030 United Nations Agenda for Sustainable Development from which the need for public policies and gender equality laws is emphasized in order to contribute from a transversal perspective to the improvement of coexistence and to a fairer and more sustainable world [4,5].

Despite the advances in ‘sexual orientation and gender identity rights’ in today’s democratic societies, as proposed by UNESCO (United Nations Educational, Scientific and Cultural Organization [UNESCO] [6,7,8], there is still resistance to achieving real equality between men and women (UNESCO, [6,7,8] and full attention to emotional-sexual, physical and gender diversity. UNESCO [7,8] has done so much, recently, in this space. There is acknowledgement of the globally recognized, recent and relevant UNESCO contributions on LGBTIQ themes. One example is its technical advice to states and researchers to explore and measure school violence and resilience for LGBTIQ youth (UNESCO, 2019). Another example is its support for research training to states and researchers to explore resilience for LGBTIQs, for example UNESCO [7]. To advance toward a more just and equitable society, our main objective must be to work toward gender equality and sexual, body, and gender diversity. Education is an instrument of social transformation that can help prevent several types of social disadvantage [9]. This is especially true when our current laws and other less recent educational reforms do not consider these values to form part of curricular content or, therefore, of the teaching-learning process. However, a good education should not be solely content-based, but should encourage students to incorporate gender equality and coeducation as a consolidated part of their identities, helping them to assimilate these values as a meaningful part of their lives through specific learning activities. Thus, besides providing students with a conceptual understanding of the importance of these issues [10], educational activities and events promoting sexual, body, and gender diversity should be designed to help them apply these issues to their own personal lives.

There is a contradiction between the progresses achieved within the legal framework and the daily social practices that continue to perpetuate prejudices and stereotypes leading to discrimination, bullying, work stress, burnout and multiple physical, psychological, economic, labor, health, and educational forms of violence towards group diversity. LGTBIQ people find their rights in retrogression compared to those of heterosexual people. In the world of work there is a tendency to reduce nonhegemonic identities—those not conforming to the heteronormative model—to a state of invisibility. This invisibility is linked to the sexist use of language, the annulment of the subjects, and the silencing of their identity. It is in this way that language generates violence with this invisibility, which leads to high levels of stress and mental exhaustion in the workplace [11]. There is a presumption in workspaces of mandatory and desired heterosexuality as the hegemonic and standard orientation for life in society. Any other non-heterosexual sexual orientation is thus belittled, stigmatized, and denied [12].

This is how discrimination against the LGTBIQ collective expresses what are known as LGTBI-phobic attitudes. LGTBIQ-phobia responds to the socio-cultural exclusion of those dissident minorities that do not respond to the expected gender mandates in a heterosexist society [13]. This leads to situations of labor discrimination against LGTBIQ workers who express their sexual or gender identity [13]. According to data, LGTBIQ people can yield a high performance in order to achieve equal opportunities and the maintenance of good mental health in the workplace for all people, regardless of their sexual-affective orientation [13,14]. LGTBIQ people are subject to higher levels of bullying, job stress, burnout, or reduced job opportunities than heterosexual people [13,15].

In the world of work, LGTBIQ visibility leads to higher levels of bullying and burnout than occurs with heterosexual employees. Moreover, LGTBIQ persons, on account of not responding to compulsory heterosexuality, are exposed to verbal violence (insults, ridicule, threats), situations of mistreatment, and workplace harassment by peers or management teams [16] That is why LGTBI workers decide to hide their sexual orientation to avoid situations of rejection, exclusion, mistreatment, or nonguarantee of equal labor rights [16].

The struggle to achieve equal opportunities and good mental health for all, regardless of sexual orientation, is not yet over, because the LGB (lesbian, gay and bisexual) community continues to experience fewer employment opportunities and higher levels of bullying and work-related stress and burnout than heterosexuals [13]. Thus, bisexual women experience more bullying and burnout that their heterosexual counterparts, bisexual men experience more burnout than heterosexual men [17], and gay men experience more burnout than either their heterosexual or their female LGB peers. In turn, the latter experience more burnout than their heterosexual counterparts [18]. The above leads to a psychological state of exhaustion and ineffectiveness in the work environment among LGTBIQ persons [19]. For example, gay teachers must show that, in addition to having a non-heterosexual sexual orientation, they can fulfill their teaching role as effectively as any other education employee, thus suffering from the hyper-sexualization of their identity. Likewise, lesbians are exposed to triple discrimination in the world of work: for their status as workers, for perceiving themselves as lesbians, and for being women [13].

Burnout is understood as “a state of chronic stress, experienced by healthcare workers in relation to patient care, which was characterized by suffering a state of exhaustion, emotional distancing, and loss of confidence in their ability to effectively perform the job” (Maslach, [20], cited in [21] p.444). [21]. As shown in this study [21], it can be noted that the burnout index in the academic context has increased. Burnout has been defined from Maslach’s consolidated model Maslach and Jackson, [22], as a behavioral manifestation of work stress, which constitutes a three-dimensional syndrome characterized by emotional fatigue (EC), depersonalization (DP) in dealing with clients and users, and difficulty in personal achievement/realization (PR) [23] in which it is expected that gender stereotypes may have negative consequences on the greater or lesser well-being in the workplace [24]. Teaching staff often present with burnout [25], which is associated with symptoms of psychological (everyday stress and anxiety), psychosocial (adjustment problems), and somatic (physical fatigue) distress [26]. These symptoms are seen more frequently in LGB people, and therefore burnout prevention is fundamental to ensure the mental health of LGB people working in any occupation.

Work burnout generates a series of symptoms that are more commonly observable in LGTBIQ people and, therefore, its prevention is essential for the mental health of LGTBIQ people. Post-identity results in sexual dissidences; that is, non-heterosexual people suffer from the inhibition of their emotions due to the pressure of the patriarchal ideological model and its normative gender mandates, which assign binary sexist roles in society. Inhibition causes depression, lowered self-esteem, and other long-term adverse health effects. Sexual minorities—in other words, non-heterosexuals—may experience burnout as a result of sexist stereotypes and gender roles that establish normative expectations about how people should behave. When LGB people transgress these norms, they are vulnerable to increased burnout and more dissatisfaction with their work due to the violence that may be inflicted by their heterosexual peers [27].

LGTBIQ-phobia is the rejection and discrimination of the element in society that is nonhegemonic in its sexual orientation, leading to subtle or explicit manifestations of physical, psychological, verbal, sexual, or labor violence [28]. That is why it is necessary that graduate degrees incorporate the gender and sexual affective diversity perspective into their training plans and programs in order to promote the acquisition and development, not only of conceptual competencies but also of procedural and psycho-emotional attitudinal skills related to interpersonal and intrapersonal capacities, including those that contribute to educating students and future teachers for the improvement of coexistence and prevention of violence [29]. Consequently, it is necessary to promote the development and acquisition of inter- and intrapersonal skills in undergraduate students by educating for social harmony, equality, solidarity, and attention to diversity, and transversally deconstructing gender roles and sexism in society [30,31].

Feminism may be understood as a process of renegotiating the terms of the social gender contract or, perhaps, as a process of modification and readjustment of the bases of gender domination established in society and in education [32]. At an international level, the 1989 appearance of the magazine Gender and Education (London) marked a milestone in the area of gender studies in education and opened the investigative debate of the GS’s (gender studies) in education [33,34].

That is why feminism as a tool for analyzing educational reality allows us to visualize the asymmetries of power and gender inequality between men, women, and the diverse population. Following Foucault’s methodological principle [35], we can state that feminism is a device to make people see and make them speak on the basis of our insertion in the cultural system.

Educational changes at an international level involve the transition from an education focused on the acquisition of knowledge to an education based on emotional skills and human values [36].

Emotions are key constructs related to psychological well-being, attitudes, and satisfaction with life, according to classical theoretical models such as that of Goleman [37], Mayer et al. These models propose that emotional/psychological skills may be divided into two poles inside a continuum: (a) the positive side includes elements such as emotional intelligence, self-esteem, resilience, self-efficacy, and empathic attitudes; and (b) the negative side includes symptoms such as anxiety and stress [38,39,40,41,42]. From this psychological perspective, specifically from the EuroPsy model for the development of standards for high-quality professionals, and from other frameworks, such as the European Higher Education Area [43] and the Organization for Economic Cooperation and Development, the development of social-emotional competencies is advocated, which may include socially responsible attitudes, emotional management, problem solving, and education for the improvement of coexistence, which may include the promotion of positive attitudes towards affective-sexual and gender diversity. It can be considered that the development of these emotional skills of emotional intelligence, empathy, self-efficacy, of the capacity of resilience in the face of adversity and prevention of stress can contribute to the improvement of coexistence in general and favor the development of attitudes towards diversity and gender equality. In that sense, there are still few studies focused on analyzing the specific relationships between the variables evaluated in this work in the university environment and in the workplace.

From the socio-educational models, it is important in this area from educational environments and institutions to work from the prevention of gender stereotypes [44,45]. In the same vein, from a psychological perspective, empirical research finds out about these attitudes towards sexual and gender equality and diversity and specifically how gender stereotypes determine positive or negative attitudes towards lesbians and gays [46].

From the so-called social role theory [47] it can also be pointed out that the teaching of specific cultural values, beliefs, and gender role norms of each socio-historical context influence positive and negative attitudes towards homosexual people. This also leads to judging whether certain positions are more or less appropriate for men or women according to these gender stereotypes and requirements of a given task or job [48]. It is precisely the training of the emotional dimension in the already-indicated aspects of regulation of negative emotions and the promotion of emotional intelligence, empathy, and resilience on the positive side that can favor a lower stress degree and emotional exhaustion in the labor environment [49].

A recent study [50] suggests that homophobia is rooted in this system of traditional values and stereotypes that challenge gender equality. This does not mean that many people support gender equality even if they do not personally identify themselves as feminists [51].

More studies are still needed to deepen attitudes towards gender equality in different groups, and even more so if we try to examine their relations with the constructs addressed in this work, and especially from an inclusive perspective that captures all the diversity of the LGTBIQ+ collective.

Below are the results found from some of the studies located in the search carried out.

The research carried out by Santona [50] found that men and people with less direct contact with homosexuals have more negative attitudes towards homosexuals than women, which can be considered consistent with what is shown in the theoretical models mentioned above regarding the importance of the socio-cultural context.

Considering the importance of that context, some recent studies [52] show how trans people have to face even more barriers and discrimination in some settings, such as in gender-segregated settings where these people more frequently experience negative attitudes, intolerance, and discrimination. To this situation can be added the difficulty of accurately assessing these types of attitudes since most existing instruments for the assessment of more egalitarian gender role attitudes do not capture all the diversity within nontraditional attitudes; a binary model predominates and not all possible social roles are considered, and a ceiling effect may predominate, derived among other things from previous biases [53]. In this sense, we can also point out the relevance of studies such as the one presented in this work to generate awareness of the need to address these issues from a public health policy, since it may seem that in current democratic societies it is no longer necessary to be concerned about issues related to gender inequality [54] and to pay attention to diversity with the enormous implications that can be derived from this from both a theoretical and practical point of view.

Another recent study [55] analyzed by telephone interview a sample of Chinese adults’ attitudes about social acceptance, discrimination protection, and marriage equality for gay/lesbian people finding that a majority of participants supported homosexuality and protection against discrimination, but most showed opposing attitudes toward same-sex marriage. Having more gay/bisexual friends or coworkers was found to be an important variable influencing support for homosexuality and acceptance of protection against discrimination.

Other recent research [56] evaluated in a sample of 120 university students the acceptance and attitude toward the LGBT community finding a significant relationship between acceptance and attitude based on gender among students. It reveals the existence of attitudes of tolerance, respect, and acceptance towards this community in these participants. However, we are aware that gender stereotypes are still present and impede sexual and gender equality and diversity in terms of, for example, the social positions reached by women in the workplace, salary, and other barriers [57] with the negative consequences on well-being at work that can result from this [24].

At present, gender bias seems to continue to affect the gender gap in STEM (science, technology, engineering or mathematics) careers [58]. The development of public gender equality policies is essential for an increasingly inclusive and sustainable society in coherence with the 2030 United Nations Agenda for Sustainable Development [4]. This requires this type of work to evaluate aspects related to sexual and gender diversity as well as the variables that can have an influence or a relevant role.

The influence of resilience, self-efficacy, self-esteem, emotional intelligence, empathy, and daily stress on the predisposition of attitudes towards sexual orientation and gender identity rights is considered to constitute an important aspect of active and responsible citizenship. Its acquisition favors a better adaptation to the social and work context and a greater ability to cope with everyday stress [59,60]. This work emphasizes the development of variables such as resilience, self-efficacy, self-esteem, emotional intelligence, empathy, and daily stress in relation to the predisposition of attitudes towards a work environment of cooperation, communication, and nonviolence and equal opportunities. That is why many companies incorporate equal opportunity plans for men, women, and people of diversity. The lower the level of emotional competency, the greater the failure of individuals, groups, and companies in relation to productivity [61]. The DeSeCo (Definition and Selection of Competencies) European project of the OECD (Organisation for Economic Cooperation and Development) defines emotional competencies of vital importance to prevent job burnout [62,63].

The relationship between burnout and the variables of resilience, self-efficacy, self-esteem, emotional intelligence, empathy, and everyday stress can be divided into (1) variables with a directly proportional relationship (i.e., as these variables increase, so does burnout), and (2) variables with an inversely proportional relationship (i.e., as these variables decrease, burnout increases, and vice versa). Everyday stress belongs to the first category, because as it increases, so does burnout, whereas resilience, self-efficacy, self-esteem, emotional intelligence, and empathy are inversely proportional to burnout because as they increase, the latter decreases, and vice versa. Thus, everyday stress is one of the factors that predispose to burnout [27]. LGTBI-phobia reveals the violation of human rights (exclusion, violence, hatred) since it prevents the exercise of these rights among those with different sexual orientations. Previous studies have also indicated that the psychological variables of self-efficacy, emotional intelligence, resilience, and everyday stress are associated with burnout [64]. There are attributes for the development of resilience against work burnout. Thus biological, psychological, and attitudinal attributes are included along with a support system: family, friends, educational institutions, and community.

The capacity for empathy towards LGTBIQ students predisposes towards nonracist, nonxenophobic, and nonsexist attitudes as well as a greater predisposition towards equal opportunities among men, women, and the diverse population [65].

Moreover, there are studies stating that people in the LGB community who develop resilience are able to face this rejection with high levels of self-esteem [66]. Thus, it is pertinent to determine not only the factors that contribute to increasing levels of emotional distress but also those that contribute to the resilience and reinforcement of the LGTBIQ identity in difficult situations [67].

Five factors that influence the resilience of LGB youth were identified: (1) being part of a social movement and understanding the impact of homophobia and other forms of oppression; (2) an ability to confront internalized homophobia; (3) expression of emotions (anger, sadness associated); (4) family acceptance and support; (5) contact with other members of the LGTBIQ community [65]. There is a correlation between self-esteem, resilience, and revealing one’s non-heterosexual identity. It has been noted that young people who feel proud of who they are and manage to make their sexual orientation publicly visible are more resilient. Higher levels of resilience lead to fewer negative emotional signs of stress, anxiety, depression, or anger, and higher levels of emotional health and self-efficacy [65].

The foregoing suggests that favorable attitudes toward equality will help construct a society free of gender roles, and that this is an essential task in order to prevent job dissatisfaction, burnout, and other associated problems. An essential first step toward achieving this goal is to study university students’ attitudes toward gender equality in relation to the variables predisposing to burnout, and in this regard it is particularly important to study teaching students since they are at risk of high levels of burnout.

In accordance with the University of Granada’s Plan for Equal Opportunities between Women and Men, the university sector constitutes one of the main agents of change as regards attention to diversity and education for gender equality. The present study formed part of an advanced teaching innovation project entitled “Cross-disciplinary education for sexual, body, and gender diversity” (Code 419, Calls for Teaching Innovation and Good Practices Projects, Plan FIDO UGR 2018-2020) [30]. The aim of this study was to assess attitudes toward inclusion and gender equality in a sample of trainee teachers undertaking a degree in primary education.

### 1.1. Research Hypothesis

The research hypothesis of this study is as follows: attitudes toward equality in undergraduates are significantly related to the variables of resilience, self-efficacy, self-esteem, emotional intelligence, empathy, and everyday stress.

### 1.2. Aims

Therefore, the aim of the present study was to analyze attitudes toward equality in undergraduate university students enrolled in teaching degrees, and to determine the possible relationship between these and the variables of resilience, self-efficacy, self-esteem, emotional intelligence, empathy, and everyday stress. Our study rationale was that it is important to prevent burnout, especially in LGB people; that teaching staff often present the symptoms associated with this condition; and that it is necessary to involve the entire community as well as interpersonal and social networks in order to prevent burnout, since it is society which perpetuates the gender roles and sexist roles that negatively affect LGB people.

## 2. Materials and Methods

### 2.1. Design

This was a cross-sectional, observational, descriptive study. An ex post facto design was employed. For this study, a cross-sectional, nonexperimental research design was selected involving convenience sampling of student participants. Participants were students recruited from the Faculty of Educational Sciences, and were taking the subjects Psychology of Education and Psychoeducational Attention to Students with Special Educational Needs and were asked to complete a series of self-report scales with a Likert response format. The data were analyzed using correlation and multiple regression statistics.

### 2.2. Participants

Participants comprised 170 university students aged between 18 and 26 years old, who were undertaking a degree in primary education at the University of Granada. The students were in their 1st and 4th years of the degree, delivered in the Faculty of Educational Sciences, and were taking the subjects Psychology of Education and Psychoeducational Attention to Students with Special Educational Needs. Of the total sample, 67% were women and 33% men; ages ranged from 18 to 26 years old, with a mean age of 20.86 years; and most were single. Convenience sampling was used to recruit participants. The inclusion criteria were: being a university student. The exclusion criteria included: (a) not completing each of the questionnaires provided administered; (b) not being a full-time student, having recognized part-time student status, and/or having requested a single assessment; and (c) being a student with special educational needs. We calculated the sample size required to detect this size effect in the sample. This was carried out using G*power 3.1 software (version 3.1, Institut für Experimentelle Psychologie, Düsseldorf, Germany). This calculation demonstrated that a sample size of 140 university students was needed to provide a confidence interval of 95%, with a power of 95%, assuming a bilateral significance level (α) of 0.05. The present study protocol was approved by the Ethics Committee of the University of Granada (Granada, Spain, 1574/CEIH/2020).

### 2.3. Instruments

Adaptation of School Doing Gender/Students—Scale of Student Attitudes toward [68]. This instrument assesses student attitudes toward coeducation and the construction of a new gender culture in educational institutions based on equality between people. It is scored using a 5-point Likert-type scale where 1 = strongly disagree and 5 = strongly agree. Previous studies that have administered this scale have presented the individual results for each item or have grouped them according to the following three levels: sociocultural level (e.g., “It is normal for boys and girls to play the same games”); relational level (e.g., “A girl should not go out with any boys other than her boyfriend”), and personal level (e.g., “I think women should not be bullfighters or soccer players”).

Modern Homophobia Scale [69]. This instrument consists of 22 items in the subscale homophobia toward gay men (MHS-G) and 24 items in the subscale homophobia toward lesbians (MHS-L). It is scored using a Likert-type scale where 1 = strongly disagree, 2 = somewhat disagree, 3 = neither agree nor disagree, 4 = somewhat agree and 5 = strongly agree. It measures subtle homophobic attitudes toward gays and lesbians, and has shown satisfactory psychometric properties when administered and validated in educational contexts [70]. A higher score indicates a more positive attitude to homosexuality, evidenced in more positive attitudes toward lesbians and gays. A total score can be obtained for each subscale, or alternatively, the items from each can be grouped into two factors at personal level: personal discomfort (e.g., “I wouldn’t mind going to a party that included lesbians”) and deviance/changeability (e.g., “Male homosexuality is a psychological disease”); and institutional level, termed institutional homophobia (e.g., “School curricula should include positive discussion of lesbian topics”).

Resilience Scale [71]; 14-item Spanish version of the Resilience Scale (RS) by Wagnild [72]. This instrument consists of 14 items scored from 1 (strongly disagree) to 7 (strongly agree). It measures the degree of individual resilience, considered as a positive personality trait that allows individuals to adapt to adverse situations related to personal competence (self-confidence, independence, decision, ingenuity, and perseverance) and accept themselves and life (adaptability, balance, flexibility, and a stable perspective on life).

Rosenberg Self-Esteem Scale (RSES [73]. This scale was administered to analyze self-esteem in terms of participants’ positive self-assessments. It is a self-report scale that measures global self-esteem. The first five items presented here measure the respondent’s positive self-assessments and the following five items measure his or her negative self-assessments. Items are scored using a 4-point Likert-type scale where 1 = strongly disagree and 4 = strongly agree. The instrument has been widely administered, is one of the most used frequently used tools in psychology to assess this construct in this population and shows satisfactory reliability and validity (see e.g., Baños & Guillén [74].

Trait Meta-Mood Scale (TMMS-24) [75]. Emotional intelligence (EI) was measured using the TMMS-24, an adaptation to Spanish of the Trait Meta-Mood Scale by Salovey, Mayer, Goldman, Turvey, and Palfai [76]. This assesses meta-knowledge about emotional states and is scored using a Likert-type response scale from 1, “strongly disagree”, to 5, “strongly agree”. It has three dimensions, each with 8 items: (1) attention to feelings (AT), which measures the extent to which a person experiences and expresses emotions correctly (e.g., “I can always say how I feel”); (2) clarity of feelings (CL), which measures the extent to which a person accurately understands their emotions (e.g., “I can come to understand my feelings”); and (3): mood repair (RE), which measures the extent to which a person can regulate their emotions (e.g., “When I am angry, I try to change my mood”). The instrument has shown a high reliability in its three dimensions, obtaining a Cronbach’s alpha of 0.88 for attention to feelings, 0.78 for clarity of feelings, and 0.71 for mood repair.

Test of Cognitive and Affective Empathy–TECA [77]. This instrument measures cognitive-affective skills related to the level of empathy, and consists of 33 items in four dimensions that are scored using a Likert scale from 1 (totally disagree) to 5 (totally agree). The cognitive domain includes the dimensions of perspective taking (capacity for tolerance, communication, and personal relationships) and emotional understanding (ability to recognize and understand emotional states, intentions, and impressions of others), while the affective domain includes empathic stress (connection with other people’s negative emotional states) and empathic joy (ability to share other people’s positive emotions). The instrument shows satisfactory reliability and validity, and internal consistency for the present sample ranged between 0.77 and 0.86.

Perceived Stress Scale (PSS) by Cohen, S., Kamarck, T., and Mermelstein, R. [78], adapted by Remor. This self-report instrument measures perceived stress in the previous month. It consists of 14 items scored using a 5-point scale (0 = never, 1 = almost never, 2 = sometimes, 3 = fairly often, 4 = very often) and has been administered in numerous studies [79,80].

### 2.4. Procedure

The questionnaires were individually administered at the start of morning classes. Subjects were informed that study participation was voluntary and that all information obtained from the questionnaires would be treated as confidential. This study received approval from the ethics committee for research on humans at the University of Granada. The mean time for questionnaire administration was 15 min.

### 2.5. Data Analysis

SPSS software (version 22.0, IBM Corp., Armonk, NY, USA) was used for the statistical analysis. All statistical analyses were performed using SPSS v. 20.0. First, the descriptive analysis was calculated and the normal distribution of variables was confirmed by means of the Kolmogorov-Smirnov test. A scatter diagram was used to verify compliance with the assumptions of linearity and homoscedasticity and determine whether to apply parametric or nonparametric tests. Pearson’s correlation coefficient was used to determine the association between attitudes toward gender equality and homophobia and each of the psychoeducational variables of resilience, self-esteem, emotional intelligence, empathy, and everyday stress. Normality of residuals, homogeneity of variance for residuals and linearity of data were examined before completing the regression model. The data met all the assumptions required to carry out the multiple linear regression analyses. Multicollinearity was avoided by selecting a stepwise method in the regression model. A *p* < 0.05 was used as the significance level in the study.

Multiple linear regression analyses were conducted to determine the relationships between attitudes toward gender equality, homophobia, resilience, self-esteem, emotional intelligence, empathy, and everyday stress in university students. A value below *p* < 0.05 was considered statistically significant in all cases.

## 3. Results

Table 1 gives the descriptive statistics for the study variables.

Below, Table 2 presents analyses of differences by sex for the variables attitudes toward gender equality and homophobia.

The results show statistically significant differences by sex in the score for the variable attitudes toward gender equality and homophobia. Women obtained a higher mean score than men for all levels or dimensions of attitudes toward gender equality; however, statistically significant differences by sex were only observed at sociocultural and relational levels.

As regards to the variable of homophobia, we found statistically significant differences by sex for the factors of personal discomfort and deviance/changeability in the homophobia toward lesbians subscale, whereby women obtained a higher score for the factor of personal discomfort whereas men obtained a higher score for the factor of deviance, indicating more positive attitudes toward lesbianism. A higher score in the Modern Homophobia Scale indicates a more positive attitude to homosexuality, evidenced in more positive attitudes toward lesbians and gays.

The dependent variables (each dimension on the attitudes toward gender equality scale and Modern Homophobia Scale) exhibited a normal distribution. After testing the normality of the residuals in the regressions, it was confirmed that the observed residuals were normally distributed. The independence of data for all regressions performed for each dimension was confirmed. There was a linear relationship between the independent variables and the dependent variables. Homogeneity of the residuals’ variance was not violated and the data met the assumption of homoscedasticity.

Table 3 shows the correlations between the variables of positive attitudes toward gender equality and homophobia and the other study variables. We found statistically significant associations between students’ attitudes toward gender equality at all three levels (sociocultural, relational, and personal) and the variable of stress burnout.

At the personal level of students’ attitudes toward gender equality, we found positive correlations between this level and the total score for the variable of resilience and with its factor of personal competence.

In reference to homophobia toward gays (positive attitudes), we observed positive correlations between the three factors of the subscale and the variable of empathy in its emotional dimension (empathic joy), while for the factor of the homophobia toward gays subscale we also found statistically significant positive correlations with both subscales of the cognitive dimension of the variable of empathy, i.e., perspective taking and emotional understanding.

Table 4 gives the total score obtained for study variables in regression analyses of “attitudes toward gender equality”, while Table 5 gives the results obtained in regression analyses of significant models of items of the variable “attitudes toward gender equality”.

Stress and “homophobia toward lesbians” were significantly associated with the dependent variable, predicting 21% of the total variance (*r*^2^ = 0.209, *F*(2,168) = 1.946, *p* < 0.001) in students’ positive attitudes toward gender equality.

In general terms, attitudes toward gender equality influenced and showed statistically significant relationships with the variables of everyday stress, homophobia toward lesbians, emotional intelligence, resilience, and empathy (Table 5, Table 6 and Table 7), and also therefore with burnout. There was no collinearity between the variables included in the regression model.

At the sociocultural level of attitudes toward gender equality for the item “Clothes and pink things are more for girls than for boys”, everyday stress, homophobia toward lesbians, and cognitive empathy (emotional understanding) were significantly related to the dependent variable, predicting 22.5% of the total variance (*r*^2^ = 0.225, *F*(3,168) = 2.758) for that item. Still at sociocultural level, for the item “Gay and lesbian people are as normal and respectable as I am”, everyday stress, homophobia toward lesbians, and resilience were significantly related to the dependent variable, predicting 18.2% of the total variance (*r*^2^ = 0.182, *F*(3,168) = 2.117) for that item.

At the relational level of attitudes toward gender equality for the item “Men are always stronger than women”, everyday stress, emotional intelligence (attention to feelings), and cognitive empathy (emotional understanding) were significantly related to the dependent variable, predicting 17.1% of the total variance (*r*^2^ = 0.171, *F*(3,168) = 1.958) for that item. For the item at relational level “It is easier to insult a homosexual than a straight man”, only emotional empathy (empathic stress) was significant in the model and related to the dependent variable, predicting 17.3% of the total variance (*r*^2^ = 0.173, *F*(1,168) = 1.971) for that item.

At the personal level of attitudes toward gender equality for the item “Women who dress like men bother me”, everyday stress and homophobia toward lesbians were significantly related to the dependent variable, predicting 17.5% of the total variance (*r*^2^ = 0.175, *F*(2,168)=2.019) for that item. For the item at personal level “I like the fact that only my father works outside our home”, everyday stress and homophobia toward lesbians were significant in the model and related to the dependent variable, predicting 17.1% of the total variance (*r*^2^ = 0.171, *F*(2,168)=1.958) for that item. For the item that also forms part of the personal level, “I think a kitchenette or a doll would be a good toy for boys or girls”, only homophobia toward lesbians was significant in the model and related to the dependent variable, predicting 16.6% of the total variance (*r*^2^ = 0.166, *F*(1,168)=1.894) for that item. Our results therefore indicate a significant relationship between attitudes toward gender equality and variables that predispose to burnout.

## 4. Discussion

The university environment is one of the main agents for promoting attention to diversity and gender equality education, in line with the University of Granada’s Plan for Equal Opportunities between Women and Men.

This study forms part of the advanced teaching innovation project entitled “Cross-disciplinary education for sexual, body, and gender diversity” (Code 419, Call for Teaching Innovation and Good Practices Projects, Plan FIDO UGR 2018–2020) [30]. The aim of this study was to assess attitudes towards inclusion and gender equality in a sample of trainee primary school teachers, in relation to burnout-related variables.

The results obtained show levels of sexism, homophobia, lesbophobia, biphobia, and transphobia, with particular determining importance being attached to the variables contemplated for measuring the occurrence of indicators in relation to the increase or decrease of burnout, as well as to the correlation with psychoeducational variables evaluated in this study. Sexism towards people of diversity who differ from the heteronormative model is still present in the university world, helping to reinforce the glass ceiling for this group in socio-workspaces. That is why, in 2007, the agenda of the International Labor Organization established sexual orientation among the new forms of discrimination. And the ILO (International Labor Organization) [81] confirmed that dealing with sexual orientation and gender identity in the world of work is a pending objective for non-discrimination [82].

The moral and ethical skills that professionals deploy in their work plays an essential role in developing the abilities of future professionals. However, organization managers have reported that new graduates are undertrained in these skills; as evidenced by their perception that ethical skills are of secondary importance compared to other generic skills [83].

This lack of training among students and teaching staff alike is even greater in terms of knowledge regarding education about gender equity and equality and attention to sexual, body, and gender diversity, or sexual orientation. It is important to appreciate the intentional nature of gender equality in order to raise awareness of the need for teaching staff to promote it through educational activities, training courses, and/or projects such as the one presented here.

Various authors [84,85] have highlighted the need to include this topic subject. At the personal level of attitudes toward gender equality for the item “Women who dress like men bother me”, everyday stress and homophobia toward lesbians were significantly related to the dependent variable, predicting 17.5% of the total variance for that item. For the item at personal level “I like the fact that only my father works outside our home”, everyday stress and homophobia toward lesbians were significant in the model and related to the dependent variable, predicting 17.1% of the total variance for that item. For the item that also forms part of the personal level, “I think a kitchenette or a doll would be a good toy for boys or girls”, only homophobia toward lesbians was significant in the model and related to the dependent variable, predicting 16.6% of the total variance for that item. Our results therefore indicate a significant relationship between attitudes toward gender equality and variables that predispose to burnout. It is necessary to implement specific actions to promote knowledge and teaching about issues related to an inclusive education for affective-sexual diversity. This highlights the importance of working to raise the visibility of diversity visible within educational-domain contexts [86] as well as the promotion of positive attitudes within the realm of sports [87]. Other recent research with university students [88,89,90] has also focused on changing negative attitudes and promoting respect towards affective-sexual diversity.

It is regarded as necessary to include content on gender and sexual diversity in the training plans for educational sciences studies. The inclusion or noninclusion of sexual affective diversity is an indicator of the quality of educational systems. Gender equality is required to preside over interpersonal relationships between teachers and educational administrative institutions. Therefore, one of the lines of action for teachers as “agents of equality” within the framework of formal education is to develop good co-educational practices, that is, to implement egalitarian actions, behaviors, and practices (learning how to do) that contribute to the elimination of a patriarchal scientific and didactic model. Requesting the modification of the sexist behavior practices we have been programmed with can be complex, but it is important to be able to adapt legal regulations in daily classroom practices in order to achieve a predisposition of attitudes towards gender equality both in the university training field and in the professional-work environment [91].

At this level, we propose that the educational community carry out a comprehensive transformation in teaching-learning processes and in the educational curriculum, cross-sectionally incorporating the gender and sexual affective diversity perspective in order to prevent work and social burnout. On the basis of feminism, we propose a paradigm shift aiming at greater teacher involvement in the pedagogical-didactic implementation at a conceptual, procedural, and attitudinal level of what is sanctioned in the regulations of public policies of Institutional Equity for the Prevention of Gender Violence in the framework of Plans for Equality between Men, Women, and Populations of Diversity in the university environment and teacher-student training [91].

The data obtained are considered relevant for the development of socio-emotional skills and attention to diversity that can contribute to the improvement of coexistence with the prevention of stereotypes and awareness of the importance of the variables of resilience, self-efficacy, self-esteem, emotional intelligence, empathy and daily stress in future programs

The objective is to contribute to the construction of a full and equal university and post-employment training, respectful of sexual and gender affective diversity, in order to evaluate the occurrence of these variables in future psychoeducational evaluation and intervention programs for the development of socio-emotional competencies bearing on coeducation for gender equality among men, women, and the LGTBIQ population.

The results show the need to generate scientific evidence of the damage caused by the lack of predisposition towards gender equality and sexual affective diversity. The less the prevention and promotion of rights regarding the LGTBIQ group, the higher the levels of violence and burnout in socio-work fields.

The obligation to remain in the closet alongside LGTBI-phobia causes LGTBI workers damage to their health, such as burnout, low self-esteem, self-exclusion, and depression. And in extreme situations, suicide. The incorporation of the perspective of affective, sexual and gender diversity in the agenda of university and business public policies guarantees an educational, training, and work environment that favors its development as well as access to social and labor benefits.

### Limitations and Future Research

Some limitations should be considered in the present study. First, the study employed a cross-sectional design and this design does not allow establishing causal relationships between the investigated variables. In the future, it is necessary to perform studies with a longitudinal design to evaluate the directionality of the relationships (cause–effect relationship).

Second, in future studies it is necessary to continue applying even more inclusive instruments that capture the diversity within nontraditional attitudes and that take into account an evaluation of negative and positive attitudes in a broader group that takes into account greater diversity within the LGBTI+ community, including trans, intersex, and gender nonconforming individuals. Likewise, it is necessary to take into account the intersectionality of the LGBTI + community by including a series of other variables such as sex, age, heteronormativity/homonormativity, or the adaptation of LGTBI + people to the norms of gender, class, or social status, and especially other types of factors that may consider the risk of poverty or social exclusion, and opportunities for access to employment and education.

Third, it would be interesting if other studies expanded the sample to include students’ data from several educative centers, degrees, from other contexts, countries and cultures, as well as a wider Spanish population in order to reach a greater segment of the reference population, for which it is necessary to continue with the evaluation and adaptation of instruments that affect the availability of more data on the aspects related to this study such as resilience, stress, and attitudes towards equality, among others. Findings are relevant to our country or regional context and to UNESCO′s work in the area. The single-country study′s limitation is that we do not know the extent of other national and regional influences on resilience and other variables.

Fourth, this study offers significant findings and implications for education and workplace management policies. These findings will make it possible to have valuable information for developing psychoeducational interventions for the prevention of burnout and homophobia in educational, work, and health contexts. However, the instruments of this study were based on student self-reports which may be influenced by the social desirability of participants studied. In other investigations, multiple reporting from other educational agents could be used and they could incorporate other qualitative instruments such as observational records and interviews to collect data.

## 5. Conclusions

Education is a key factor in guaranteeing justice, social cohesion, and gender equality. Education, we believe, must be transformed “from within”, emphasizing the underlying emotion in educational processes in which teachers are the first recipients of emotional education. The greater the development of teachers’ emotional competencies, the higher the levels of mental health, prevention of burnout, and predisposition towards equality on the part of the students.

UNESCO has done so much in this space. UNESCO contributions on LGBTIQ themes are globally recognised, recent, and relevant. Here, we present the results of the relationships with the psychoeducational variables of attitudes toward gender equality, homophobia toward gays and lesbians, self-esteem, emotional intelligence, empathy, levels of stress, and resilience in university students. The data obtained will be of use for educational interventions intended to improve social harmony, especially as regards to important aspects related to gender equality, sexual, body, and gender diversity, and the prevention of burnout and disengagement due to experiences related to gender inequality, violence and lack of attention to diversity.

In conclusion, understanding the cognitive, socio-emotional, and behavioral process of this study and its associated variables is valuable, not only for clinical work but for collective change towards a society that is more receptive to sexual and gender diversity rights. Ultimately the study highlights how this knowledge can be a powerful tool in the hands of skillful teachers who then can set about effective social change.

## Figures and Tables

**Table 1:** Descriptive results (range, mean, and SD) of attitudes toward gender equality, homophobia toward gays, homophobia toward lesbians, resilience, self-esteem, emotional intelligence, empathy, and levels of everyday stress in university students.

Measurement Variables	Range	Minimum	Maximum	Mean	SD
Attitudes toward gender equality (total)	52	95	147	135.81	9.56
Sociocultural level	22	28	50	46.89	4.06
Relational level	23	27	50	47.05	4.26
Personal level	18	29	47	41.79	2.47
No Homophobia toward gays (total) (positive attitudes toward lesbians)	37	44	81	59.23	4.65
Personal discomfort	17	24	41	35.88	3.10
Deviance	10	4	14	4.74	1.85
Institutional homophobia	20	13	33	18.60	3.42
No Homophobia toward lesbians (total) (positive attitudes toward lesbians)	50	42	92	81.84	6.61
Personal discomfort	38	10	48	43.46	5.51
Deviance	12	3	15	3.47	1.87
Institutional homophobia	20	23	43	35	3.63
Resilience (total)	56	41	97	76.71	10.45
Resilience (personal competence)	41	22	63	50.13	6.96
Resilience (acceptance of oneself and of life)	16	5	21	14.85	3.04
Self-esteem	30	10	40	21.80	8.92
Emotional intelligence					
Attention to feelings	23	17	40	30.52	5.18
Clarity of feelings	25	15	40	28.72	5.98
Mood repair	28	12	40	28.73	5.73
Empathy					
Perspective taking	23	17	40	31.44	4.21
Emotional understanding	30	20	50	39.05	5.07
Empathic stress	27	13	40	28.78	5.26
Empathic joy	16	19	35	31.14	3.60
**Stress**	40	6	46	26.34	7.48

**Table 2:** Comparison of means (Student’s *t*-test) to determine differences by sex for the study variables.

Study Variables	Men		Women		Student’s *t*-Test			
	*M*	*SD*	*M*	*SD*	*t*	*df*	*p*	*d*
Attitudes toward gender equality (total)	130.90	13.51	137.81	6.31	−4.37	168	0.00	−6.91
Sociocultural level	45.14	5.33	47.54	3.25	−3.55	168	0.00	−2.39
Relational level	44.46	6.21	48.12	2.49	−5.36	168	0.00	−3.66
Personal level	41.33	3.54	41.91	2.36	−1.23	168	0.22	−0.57
No Homophobia toward gays (positive attitudes toward gays) (total)	58.55	4.26	60.01	4.67	−1.26	168	0.20	−1.46
Personal discomfort	36.20	1.90	36.36	2.53	−0.26	168	0.79	−0.16
Deviance	4.70	1.78	4.85	1.98	−0.31	168	0.75	−0.15
Institutional homophobia	17.65	3.45	18.78	3.50	−1.28	168	0.20	−1.13
No Homophobia (positive attitudes toward lesbians) (total)	80.79	6.39	82.75	5.69	−1.83	168	0.06	−1.95
Personal discomfort	42.04	5.62	44.22	4.58	−2.49	168	0.01	−2.18
Deviance	4.21	2.82	3.15	1.19	3.27	168	0.00	1.06
Institutional homophobia	34.80	3.65	35.37	3.47	−0.90	168	0.36	−0.57

*df*: degrees of freedom; *p*: level of significance; *t*: Student’s *t*-test statistic for 2 independent samples; *d*: difference in means; *M*: mean; *SD*: standard deviation; *p*: critical level of significance.

**Table 3:** Correlations between attitudes toward gender equality, homophobia toward gays and lesbians, self-esteem, emotional intelligence, empathy, levels of everyday stress, and resilience in university students.

Measurement Variables	1	2	3	4	5	6	7	8	9	10	11	12
Attitudes toward gender equality (total)	−0.10	−0.05	−0.13	−0.03	−0.00	0.05	0.08	0.00	−0.27 **	0.13	0.10	0.10
Sociocultural level	−0.08	−0.04	−0.05	−0.00	0.01	0.03	0.07	0.02	−0.16 *	0.07	0.03	0.08
Relational level	−0.09	−0.11	−0.04	−0.13	−0.04	−0.00	0.07	0.10	−0.30 **	0.11	0.02	0.10
Personal level	−0.06	−0.05	−0.13	−0.06	0.00	0.02	0.04	−0.03	−0.18 *	0.17 *	0.15 *	0.11
No Homophobia toward gays (positive attitudes total)	0.09	0.01	0.00	0.07	0.07	0.11	−0.01	0.08	0.07	0.06	0.03	0.08
Personal discomfort	0.05	0.10	−0.07	−0.03	−0.05	−0.15	−0.07	0.19 *	−0.02	0.10	0.09	0.08
Deviance	0.00	0.11	0.04	0.09	0.09	0.11	−0.02	0.17 *	0.09	0.02	0.00	0.04
Institutional homophobia	0.07	0.02	0.02	0.10	0.18 *	0.17 *	0.07	0.20 *	0.06	−0.01	−0.04	0.01
No Homophobia toward lesbians (positive attitudes total)	0.04	−0.09	−0.12	−0.06	0.07	0.03	0.14	0.02	−0.04	0.04	0.03	0.04
Personal discomfort	−0.01	−0.09	−0.14	−0.11	0.06	−0.02	0.09	0.02	−0.05	0.01	−0.00	0.03
Deviance	0.10	0.11	0.09	0.13	−0.01	0.09	−0.09	0.11	0.09	−0.04	−0.03	−0.06
Institutional homophobia	0.03	−0.08	−0.06	−0.00	−0.05	0.05	0.14	−0.03	−0.04	0.07	0.07	0.05

1. self-esteem; 2. attention to feelings; 3. clarity of feelings, 4. mood repair; 5. perspective taking; 6. emotional understanding; 7. empathic stress; 8. empathic joy; 9. everyday stress; 10. resilience; 11. resilience personal competence; 12. resilience acceptance of oneself and of life; * *p* < 0.05; ** *p* < 0.01.

**Table 4:** Regression of “attitudes toward gender equality” for the variables homophobia toward gays and lesbians, self-esteem, emotional intelligence, empathy, levels of everyday stress, and resilience in university students.

Attitudes Toward Gender Equality (total) (*r*^2^ = 0.209)						
Independent Variables	B	*CI* 95%		*β*	*SE*	*p*-Value
		Lower Limit	Upper Limit			
Stress	−0.29	0.05	0.54	0.23	0.12	0.00
No Homophobia toward lesbians (positive attitudes)	0.36	0.10	0.63	0.26	0.13	0.01

*r*^2^: regression coefficient of determination; B: regression coefficient estimates; *β*: normalized regression coefficient estimates; *CI* 95%: 95% confidence interval for B; *β*: adjusted multiple linear regression coefficient; *SE*: standard coefficient error; *p*: critical level of significance.

**Table 5:** Regression of “attitudes toward gender equality” (statistically significant items at sociocultural level) for the variables homophobia toward gays and lesbians, self-esteem, emotional intelligence, empathy, levels of everyday stress, and resilience in university students.

Attitudes Toward Gender Equality (Sociocultural Level)						
Independent Variables	B	*CI* 95%		*β*	*SE*	*p*-Value
		Lower Limit	Upper Limit			
Clothes and Pink Things are More for Girls than for Boys (*r*^2^ = 0.225)						
Stress	−0.02	−0.03	−0.00	−0.19	0.00	0.02
No Homophobia toward lesbians (positive attitudes)	−0.03	−0.05	−0.01	−0.34	0.00	0.00
Cognitive empathy (emotional understanding)	−0.03	−0.06	−0.00	−0.25	0.01	0.01
Gay and lesbian people are as normal and respectable as I am (*r*^2^ = 0.182)						
Stress	−0.02	0.01	0.03	0.33	0.00	0.00
No Homophobia toward lesbians (positive attitudes)	0.01	0.00	0.02	0.21	0.00	0.03
Resilience	−0.01	−0.01	−0.00	−0.18	0.00	0.01

*r*^2^: regression coefficient of determination; B: regression coefficient estimates; *β*: normalized regression coefficient estimates; *CI* 95%: 95% confidence interval for B; *β*: adjusted multiple linear regression coefficient; *SE*: standard coefficient error; p: critical level of significance.

**Table 6:** Regression of “attitudes toward gender equality” (significant items at relational level) for the variables of homophobia toward gays and lesbians, self-esteem, emotional intelligence, empathy, levels of everyday stress, and resilience in university students.

Attitudes Toward Gender Equality (Relational Level)						
Independent Variables	B	*CI* 95%		*β*	*SE*	*p*-Value
		Lower Limit	Upper Limit			
Men are Always Stronger than Women (*r*^2^ = 0.171)						
Stress	−0.02	−0.04	−0.00	−0.18	0.01	0.04
Emotional intelligence (attention to feelings)	0.03	0.00	0.07	0.20	0.00	0.02
Cognitive empathy (emotional understanding)	−0.04	−0.08	−0.00	−0.23	0.02	0.03
It is easier to insult a homosexual than a straight man (*r*^2^ = 0.173)						
Emotional empathy (empathic stress)	−0.03	−0.07	−0.00	−0.24	0.01	0.03

*r*^2^: regression coefficient of determination; B: regression coefficient estimates; *β*: normalized regression coefficient estimates; *CI* 95%: 95% confidence interval for B; *β*: adjusted multiple linear regression coefficient; *SE*: standard coefficient error; *p*: critical level of significance.

**Table 7:** Regression of “attitudes toward gender equality” (significant items at personal level) for the variables of homophobia toward gays and lesbians, self-esteem, emotional intelligence, empathy, levels of everyday stress, and resilience in university students.

Attitudes Toward Gender Equality (Personal Level)						
Independent Variables	B	*CI* 95%		*β*	*SE*	*p*-Value
		Lower Limit	Upper Limit			
Women who Dress like men Bother me (*r*^2^ = 0.175)						
Stress	−0.01	−0.02	−0.00	−0.29	0.00	0.00
No Homophobia toward lesbians (positive attitudes)	−0.01	−0.02	−0.00	−0.24	0.00	0.01
I like the fact that only my father works outside our home (*r*^2^ = 0.171)						
Stress	−0.01	−0.02	−0.00	−0.27	0.00	−0.00
No Homophobia toward lesbians (positive attitudes)	−0.01	−0.02	−0.00	−0.24	0.00	−0.00
I think a kitchenette or a doll would be a good toy for boys or girls (*r*^2^ = 0.166)						
No Homophobia toward lesbians (positive attitudes)	−0.04	−0.06	−0.01	−0.33	0.01	0.00

*r*^2^: regression coefficient of determination; B: regression coefficient estimates; *β*: normalized regression coefficient estimates; *CI* 95%: 95% confidence interval for B; *β*: adjusted multiple linear regression coefficient; *SE*: standard coefficient error; *p*: critical level of significance.
